# Strategy for the Maximal Use of Native Arteriovenous Fistulae for Hemodialysis

**DOI:** 10.1100/tsw.2006.171

**Published:** 2006-07-14

**Authors:** Marko Malovrh

**Affiliations:** Department of Nephrology, University Medical Center Ljubljana, Ljubljana, Slovenia

**Keywords:** arteriovenous fistula, predialysis nephrologic care, referral, hemodialysis, duplex sonography, Doppler, blood flow, resistance index

## Abstract

The long-term survival and quality of life of patients on hemodialysis is dependant on the adequacy of dialysis via an appropriately placed vascular access. The native arteriovenous fistula (AV fistula) at the wrist is generally accepted as the vascular access of choice in hemodialysis patients due to its low complication and high patency rates. It has been shown beyond doubt that an optimally functioning AV fistula is a good prognostic factor of patient morbidity and mortality in the dialysis phase. Recent clinical practice guidelines recommend the creation of a vascular access (native fistula or synthetic graft) before the start of chronic hemodialysis therapy to prevent the need for complication-prone dialysis catheters. A multidisciplinary approach, including nephrologists, surgeons, interventional radiologists, and nurses should improve the hemodialysis outcome by promoting the use of native AV fistulae. An important additional component of this program is the Doppler ultrasound for preoperative vascular mapping. This approach may be realized without unsuccessful surgical explorations, with a minimal early failure rate, and a high maturation, even in risk groups such as elderly and diabetic patients. Vascular access care is responsible for a significant proportion of health care costs in the first year of hemodialysis. These results also support clinical practice guidelines that recommend the preferential placement of a native fistula.

## INTRODUCTION

The provision of adequate hemodialysis is dependent on repeated and reliable access to central circulation. An ideal access delivers a flow rate adequate for the dialysis prescription, has a long use-life and has a low rate of complications (e.g., stenosis, thrombosis, aneurysms, limb ischemia, and infections). Although no current access type fulfills all of these criteria, the native arteriovenous (AV) fistula comes the closest to doing so. Nowadays, the most frequent problem with the AV fistula is primary failure and unsuccessful maturation with inadequate blood flow during hemodialysis treatment. The first AV fistula was created in 1966 by Brescia et al.[1]. Since then, elderly and diabetic patients have been accepted for long-term dialysis treatment and a rising proportion of patients on hemodialysis for more than 20 years have been found to have specific vascular, mainly arterial, problems[2]. A too high proportion of patients begin hemodialysis treatment with a central venous catheter because an AV fistula was not previously created or was not matured on time. At the initiation of hemodialysis treatment, 68% of patients use a dialysis catheter and 32% use another vascular access (18% PTFE and 14% native AV fistulae)[3]. The complications of central venous catheters are well known: a high rate of infection and stenosis or even thrombosis of the host vein. Recent clinical practice guidelines recommend the creation of vascular access (native fistula or synthetic graft) before the start of chronic hemodialysis therapy to prevent the need for complication-prone dialysis catheters[4,5,6]. The use of a temporary catheter, compared to the AV fistula, has been associated with a worse outcome in prevalent hemodialysis patients[3,7].

## REFERRAL TO NEPHROLOGIST

Insufficient care of patients with chronic kidney disease before end-stage renal disease (ESRD) could be one of the causes of the unacceptably high mortality rates in patients on renal replacement therapy. Several studies have reported an association between late referral and the more frequent use of temporary catheters at entry[3,4,5,6,7,8]. Late referral to a nephrologist is not the only factor contributing to the high rate of dialysis-catheter use and the low rate of AV fistula use. Evidence is also lacking on the extent to which the timing of referral to a nephrologist affects the eventual choice of initial AV fistula type. Efforts to improve the vascular access experience of patients in the initial stages of hemodialysis therapy need to focus on all persons involved in predialysis care, including patients, referring physicians, surgeons, and nephrologists. An aggressive policy of venous preservation before the beginning of any renal replacement therapy is needed. It is important to instruct the patient in time and to motivate him to preserve the forearm veins. The puncture of a vein will leave a scar. When an AV fistula is created, such scars interfere with harmonious dilation and remodeling, cause turbulent flow, and predispose patients to stenosis. The rule should be: strict avoidance of cannulation of the veins of both forearms proximal to the wrist. For vein puncture, the veins of the dorsum of the hand should be used as an alternative. If difficulties arise, the hand should be warmed in a hot bath. When unavoidable, vein puncture should be performed on the dominant arm to preserve the nondominant arm for the AV fistula, or, alternatively, the rotation of puncture sites could be used. Phlebocatheters should not be threaded to the central veins through the cephalic or basilic veins at the elbow. Instead, central vein catheters should be inserted into jugular veins (preferably on the right side). Insertion via subclavian veins is to be avoided because it frequently gives rise to subsequent stenosis. The same applies for transvenously inserted pacemakers. In cases where vein diameter/flow is the critical factor influencing the decision to use the central vein (e.g., when concentrated, potentially caustic/toxic solutions are to be infused), one should consider using the femoral veins[9]. The placement and adequate maturation of AV fistula before the initiation of hemodialysis therapy requires timely patient education and counseling, selection of the preferred renal replacement modality, selection of an access type and location, and creation of the access at least several weeks to months in advance of its expected need[10]. An early-constructed AV fistula could also have a beneficial effect on the rapidity of worsening kidney failure. In a prospective randomized study, Malovrh found, after 24 months of observation, that in the group with AV fistula, 14/30 (47%) patients began with hemodialysis, 16 patients had a mean creatinine clearance (CC) of 13.1 ± 2.2 ml/min, a mean blood pressure (MBP) of 118 mmHg, and no sign of cardiac insufficiency. In the group without AV fistula, 27/30 (90%)($p < 0.01$) patients began with hemodialysis and the central vein catheter was used as a vascular access, while in the remaining 3 patients, the mean CC was 10.7 ± 1.0 ml/min ($p < 0.01$) and the MBP was 123 mmHg (NS). There was no difference in the quality of predialysis renal care between the two groups[11].

The cost of vascular access care is high among all patients and the highest among patients in whom a graft is placed. These results support the Dialysis Outcomes Quality Initiative (DOQI) guidelines that recommend the initial placement of an AV fistula[12,13]. The cost of vascular access–related care was five times lower for patients treated with hemodialysis who began the study period with a functioning AV fistula compared to those treated with a percutaneous catheter or AV graft[14].

## EVALUATION OF THE PATIENT BEFORE SURGERY

The risk factor for primary failure is not well established, although the quality of vessels is thought to play an important role[15,16,17]. One of the most important predictors of successful AV fistula development is the ability of the arterial and venous vessels to dilate under the influence of increased shear rates — vessel remodeling[18,19]. The preoperative physical examination of the patients' forearm venous and arterial vessels includes an inspection of the vein with a tourniquet in order to induce venous congestion and the quality of the arterial pulse. In many patients, especially obese persons, the clinical detection of veins and the evaluation of their dilatation possibility are impossible. Previous insertions of central vein catheters may cause central vein stenosis and consecutive outflow problems after AV fistula construction. An atherosclerotic and calcified narrowed artery will deliver only a limited blood flow rate and will not undergo adaptive flow-mediated dilatation to deliver sufficient fistula blood volume, a prerequisite for venous dilatation and satisfactory blood flow[16,19,20]. The immediate success and flow rate of a newly constructed AV fistula was mainly dependant on arterial inflow and venous outflow. In complex cases, particularly in patients with a history of previous failed fistulae or prior vein cannulation, vein mapping using duplex sonography is an additional valuable tool. Also, in patients who previously had chronic cannulation of the subclavian or jugular veins, the central veins should be evaluated by duplex ultrasonography or venography to exclude any underlying stenosis or occlusion. It was suggested that duplex imaging be used to evaluate all patients prior to the creation of an AV fistula. Duplex scanning is a promising method for establishing certain morphological and functional parameters of peripheral blood vessels because it is noninvasive and safe, and may be used in lieu of venography and arteriography at facilities where this modality is available and reliable for venous and arterial assessment. This method has been recently used to visualize and measure arterial and venous vessel diameters, and has shown a good correlation between preoperative determination and perioperative findings[15,16,18,19,20]. Malovrh, for example, demonstrated an immediate patency rate of 92% in patients with a preoperative internal diameter > 1.5 mm in the feeding artery, as compared to a maturation rate of 45% in patients with an internal diameter < 1.5 mm. At 12 weeks, the patency rate in the two groups was 83 and 36%, respectively[16]. Increased artery intima-media thickness (IMT) is known to be a risk factor of early AV fistula failure. High-resolution ultrasonography is a simple and effective tool in measuring artery IMT. The measurement of artery IMT by ultrasonography is recommended in uremic patients who have poor vascularity, such as old age, diabetes mellitus, and severe atherosclerosis patients, before the AV fistula operation[17,18]. Besides morphological evaluation, the functional characteristics of arteries could also be evaluated by duplex sonography[16,21]. The feeding arteries dilate during access maturation. Consequently, it is obvious that not only the initial diameter, but also arterial compliance, affects access outcome. The distensibility of the arterial wall can be assessed preoperatively by evaluating the Doppler waveform in the radial artery during reactive hyperemia, induced by reopening a fist that was clenched for 2 min. The high-resistance triphasic Doppler ultrasound signal with clenched fist (regular signal of peripheral arteries) changes to a low-resistance biphasic waveform after releasing the fist, and the resistance index (RI) at reactive hyperemia can be calculated using the formula: (peak systolic velocity – peak diastolic velocity)/(peak systolic velocity). A preoperative RI of > 0.7 in the feeding artery after release of the fist indicates that arterial blood flow will not increase sufficiently so that the chance of successful creation of an AV fistula is reduced. Preoperative screening to exclude an inappropriate response to reactive hyperemia is recommended. This maneuver is especially helpful when planning the location of the initial operation, i.e., selecting the wrist/forearm or elbow region[16,18,22]. The preoperative Doppler ultrasound criteria for AV fistula outcome are: arterial luminal diameter > 1.6 mm, venous luminal diameter (without use of tourniquet) > 2.0 mm, arterial resistance index < 0.7 (at reactive hyperemia).

Routine preoperative sonographic vascular mapping results in an increase of patients with suitable veins. Many patients were found to have large-caliber veins that were simply too deep to be visualized. Malovrh[18] reported that the veins were clinically visible only in 54/116 (46.5%) patients; among the 62/116 (53.5%) patients with no visible veins, they were detected by ultrasound in 48/62 (77.4%)

patients. After AV fistula construction, the "fistula vein" under the influence of increased blood flow and intravenous pressure is dilated. This ability of the vein could be determined by measuring the increase of the vein's inner diameter (IDV) after proximal vein compression. A blood pressure cuff should be placed around the upper arm as proximally as possible and inflated at 50 mmHg for at least 2 min. The predictive value of vein diameter for successful AV fistulae is yet to be established. Vein diameters < 1.6 mm have been associated with AV fistula failure[15,18,20,22,23], while good patency rates were obtained in patients with AV fistulae that were created on the basis of a selection of adequate veins (diameter of cephalic vein at the wrist ≥ 2–2.5 mm or upper arm veins > 3 mm)[19,20,21]. In the failed group of AV fistulae, IDV increased after proximal compression by 11.8% compared to 48% in the group of successful AV fistulae[18]. On the basis of this increase, we were able to anticipate the increase of vein diameter at different intervals after construction, and predict the time of AV fistula maturation.

To determine if there is any disturbance in venous outflow, the continuity of the shape of the Doppler vein signal (DVS) and respiratory filling is used. At deep breath, the venous flow is increased because of low resistance to venous flow. If there is venous outflow disturbance (stenosis), DVS is not changed[18]. Duplex ultrasound is an accurate method for the investigation of arteries in the upper extremity with a sensitivity of 90% and a specificity of 99% for the detection of obstructive disease[20]. The use of duplex ultrasound influences the choice of access placement[18,23].

Arterial spasm in uremic patients undergoing construction of the AV fistula could be an important reason for their primary nonfunctioning, especially in the marginal quality of arteries. Ultrasonic Doppler flowmetry was used for detecting such phenomena. Arterial blood flow was significantly decreased 5 min after anatomizing the radial cephalic vein with the radial artery (14.3 ± 4.5%, mean ± SE, of the initial value; $p < 0.001$ vs. initial blood flow), indicating vasospasm[24]. Increasing the blood flow could prevent spasm of the artery. In a 2-year period, Malovrh analyzed 102 native AV fistulae constructed at the Department of Nephrology. In 58 patients, the mean IDA was 2.8 ± 0.4 mm, RI at RH was 0.62 ± 0.07, ABF 44 ± 9.2 ml/min. The primary patency rate was 96.5% (56/58). In the risk group of elderly and diabetic patients with a marginal quality of vessels, the study was performed with an expansion of blood volume to find out if this could prevent early AV fistula functioning after AV fistula construction. During the surgical procedure, 22 patients with IDA 1.8 ± 0.5 mm, RI RH 0.83 ± 0.5, received a mean 720 ml (range 320–870 ml) of plasma expander. The primary patency rate in this group was 86.4% (19/22). During surgery, 22 patients with IDA 2.0 ± 0.2 mm, RI RH 0.87 ± 0.6, did not receive plasma expander. The primary patency rate was 27.3% (6/22) ($p > 0.001$). In our study, the infusion of plasma expander increased the primary patency rate. On the basis of the morphological and functional characteristics of arteries determined by duplex sonography before surgery, the need for the expansion of blood volume could be predicted[25].

## NATIVE ARTERIOVENOUS FISTULA

Despite the increasing number of patients with diabetes, peripheral vascular disease, and those of older age, the creation of a native AV fistula is possible in the vast majority of cases[27]. The long-term survival of the radiocephalic AV fistula varies widely: 85% survival in the first year and 80% in the second year[28]. Despite the advantages of the AV fistula, early occlusion or failure to mature occurs in 10–24% of patients[29]. It is difficult to compare the failure rates as there are two different "schools of thinking". Some teams attempt AV fistula creation only when the arteries and veins are of optimal quality. If these conditions are not found at the wrist, the AV fistula is created at the elbow or the upper arm. Lazarides et al. analyzed data from 1516 patients receiving long-term hemodialysis. They suggested that an AV fistula at the elbow should be considered second best[30]. Nevertheless, an AV fistula at the wrist should always be the first choice for vascular access. Malovrh analyzed 115 patients on HD treatment. The AV fistula was constructed at the wrist in 56%, at the mid-forearm in 11%, and at the elbow in 33% of patients. The reason for a mid-forearm AV fistula was the artery in 69% and the vein in 31% of patients, and for an elbow AV fistula, the artery in 40% and the vein in 60% of patients. The elbow AV fistula is a reliable means of establishing vascular access for hemodialysis if a primary AV fistula at the wrist is technically not possible, or as a secondary procedure. In contrast, other groups, especially those using microsurgery and working in close cooperation with interventional radiologists, will attempt AV fistula creation even when venography or duplex sonography shows evidence of small vessels. The latter bears the risk of higher initial failure rates and delay in achieving a functional vascular access for the patient, but less risk of mid-term and long-term morbidity, since steal syndromes and excessive high flows are much less frequent in forearm fistulae[31].

The transposition of the autologous basilic vein to the brachial artery provides suitable vascular access in the absence of a superficial vein, owing to the fact that this vein is not usually damaged by cannulation. The procedure is usually accompanied by “superficialization” of the vein for easier cannulation. A patency rate of 75% at 8 years or 76.7, and a 49.2% patency rate for the brachial artery and the basilic vein fistula at 1 and 4 years, respectively, were reported[28,32]. In high-risk patients, the autologous transposed brachiobasilic fistula has an equivalent patency and lower complication rates than those reported for PTFE interposition grafts[33]. However, there has been no clinical trial comparing these two types of upper-arm AV fistula, so no definitive advice can be given. The selection of an appropriate AV fistula will depend on the quality and topography of vessels involved.

The creation of an AV fistula is an interdisciplinary task. In several countries, the task of coordination is delegated to a “fistula manager” who integrates the activities of the nephrologist, the ultrasonographer, the surgeon, and the interventional radiologist[26]. The creation of fistulae should be delegated to a restricted number of dedicated surgeons and/or nephrologists interested in VA construction because good results are only achieved by persons with considerable expertise and an understanding of the goal of the AV fistula.

There has been much disagreement concerning the time when an AV fistula is ready for puncture. The DOQI[34] guidelines recommend puncture after 3–4 months. According to some authors, this may be too restrictive and it is considered more rational to wait longer than 10 days, except in case of specific circumstances such as skin lesions, infection, etc.[35]. Fistula flow was measured and an immediate, tenfold and more increase of blood flow rate was found in the radial artery, which progressively increased during the first 10 days[18] and tapered off later on. Their practice is to puncture the fistula no sooner than 3–4 weeks after operation. The main reasons for this are the enlargement of the fistula vein diameter and arterialization of the fistula vein wall.

## MONITORING OF THE ARTERIOVENOUS FISTULA

Nurses should be trained to recognize fistula problems and to pay attention to the progressive increase of venous inflow pressure and postpuncture bleeding. Regular fistula inspection and physical examination of the fistula is advisable every 4 weeks[36]. The main purpose is to detect the development and progression of stenosis in time to prevent any eventual thrombosis, so that one is not forced to correct an established thrombosis surgically. The final trigger causing thrombosis is the critical reduction of arterial blood flow below 200 ml/min. Several procedures help to recognize critically low blood flow rates: auscultation (high-frequency bruits at the site of stenosis), hand elevation (collapse of the poststenotic venous segment and persisting congestion of the prestenotic segment), prolonged bleeding after removal of the needle, and elevated venous inflow pressure during dialysis treatment[28]. Complete documentation enabling systematic and continuous nursing care of vascular access was prepared for short- or long-term monitoring of all changes. The documentation consists of three documents: basic information about the patient (age, sex, primary renal disease, date of vascular access construction, location, when VA was first used, etc.), a drawing of the AV fistula (artery and vein, place of anastomosis, marked puncture spots; enclosed is also a photo of the AV fistula), and a document presenting, in chronological order, any changes in puncture spots[37]. Early diagnostic procedures could be conducted and, if needed, early corrections (surgical or radiological) could be made. These documents provide new opportunities for a long-lasting (an extensive) AV fistula presentation, and may also be used to learn something new about AV fistulae for each patient.

## CONCLUSION

It is the intention of this brief review to point out that the AV fistula should be the preferred form of hemodialysis vascular access. A multidisciplinary approach, including nephrologists, surgeons, interventional radiologists, and nurses should improve the hemodialysis outcome by promoting the use of native arteriovenous fistulae at a very early stage of chronic kidney disease. There is no single standard approach and surgical management must be individualized. One key to success is early referral and the early establishment of vascular access. Numerous clinical studies have been able to demonstrate a significant improvement in the primary patency and in the maturation of AV fistulae through the aggressive preoperative ultrasound evaluation of arterial and venous vessels. Our personal experience, as well as several registry reports, document that native AV fistulae can be established in more than 80% of patients. Regular monitoring of the fistula is also indicated, and fistula flow should be assessed in critical cases as the single most important predictor of fistula thrombosis.

## ACKNOWLEDGMENTS

The author would like to express his gratitude to Professor Daniel Batlle, Principal Editor of the Nephrology domain of *TheScientificWorldJOURNAL*, for giving him the chance to present the results of his research to the international scientific community and for handling the manuscript.

The studies conducted by the author that are mentioned in this review were supported in part by the Ministry of Education, Science and Technology (Grants L3-4486-0312 and P3-0323-0312).

## BIOSKETCH

**Marko Malovrh** is Professor of Medicine, Doctor of Science, Doctor of Medicine, Specialist of Internal Medicine and Nephrology, Head of the Center for Hemodialysis, Secretary of Vascular Access Society. Past and current research interests include dialysis, vascular access, ultrasonography, vascular access surgery, and kidney transplantation
